# Supplementary material for: Drivers and Impacts of the Record-Breaking 2023 Wildfire Season in Canada
Source: Nat Commun. 2024 Aug 20;15:6764. doi: 10.1038/s41467-024-51154-7 (PMC11335882; doi:10.1038/s41467-024-51154-7)
Supplement: Supplementary file 1 — Supplementary Information [file 41467_2024_51154_MOESM1_ESM.pdf]

Supplementary information for:

Drivers and Impacts of the Record-breaking 2023 Wildfire Season in Canada

Piyush Jain[1]
Quinn E. Barber[1]
Steve Taylor[2]
Ellen Whitman[1]
Dante Castellanos Acuna[3,7]
Yan Boulanger[4]
Raphaël D. Chavardès[5]
Jack Chen[6]
Peter Englefield[1]
Mike Flannigan[7]
Martin P. Girardin[4]
Chelene C. Hanes[8]
John Little[1]
Kimberly Morrison[2]
Rob S. Skakun[1]
Dan K. Thompson[8]
Xianli Wang[1]
Marc-André Parisien[1]

[1] *Northern Forestry Centre, Canadian Forest Service, Natural Resources Canada, 5320-122 St. NW, Edmonton, AB, T6H 3S5, Canada.*

[2] *Pacific Forestry Centre, Canadian Forest Service, Natural Resources Canada, 506 West Burnside Rd., Victoria, BC, V8Z 1M5, Canada.*

[3] *Department of Renewable Resources, University of Alberta, General Services Building, 9007-116 St. NW, Edmonton, AB, T6G 2H1, Canada.*

[4] *Laurentian Forestry Centre, Canadian Forest Service, Natural Resources Canada, 1055 du P.E.P.S., P.O. Box 10380, Stn. Sainte-Foy, QC, G1V 4C7, Canada.*

[5] *Atlantic Forestry Centre, Canadian Forest Service, Natural Resources Canada, 1350 Regent Street, P.O. Box 4000, Fredericton, NB, E3B 5P7, Canada.*

[6] *Air Quality Research Division, Atmospheric Science and Technology Directorate, Environment and Climate Change Canada, Ottawa, ON, K1V 1C7, Canada.*

[7] *Natural Resource Science, Thompson Rivers University, 805 TRU Way, Kamloops, BC, V2C 0C8, Canada.*

[8] *Great Lakes Forestry Centre, Canadian Forest Service, Natural Resources Canada, 1219 Queen Street E, Sault Ste. Marie, ON, P6A 2E5, Canada.*

Supplementary Figures and Tables

Table S1: Regional summaries for total area burned in 2023

**Region**	**2023 Area burned (ha)**	**2023 rank (1986-2023)**
Quebec	4,258,416	1
Northwest Territories	3,574,813	1
Alberta	2,735,063	1
British Columbia	2,342,294	1
Saskatchewan	1,137,683	3
Yukon Territory	379,668	2
Ontario	341,599	6
Manitoba	142,334	18
Maritimes	24,064	2
Newfoundland and Labrador	19,257	18
Nunavut	2,323	10

Table S2: Regional summaries of proportion (%) vegetation type burned in 2023, based on SCANFI forest cover class types[1].

**Region**	**Herbs/bryoids**	**Shrubs**	**Broadleaf forest**	**Conifer forest**	**Mixed forest**
Quebec	1.10	18.15	0.40	76.71	3.64
Northwest Territories	4.08	13.09	4.49	69.99	8.35
Alberta	1.07	4.20	18.46	65.00	11.28
British Columbia	1.65	2.52	6.37	82.57	6.89
Saskatchewan	1.74	2.18	5.25	83.40	7.44
Yukon	5.95	29.65	3.94	56.09	4.38
Ontario	1.29	4.32	2.12	85.93	6.34
Manitoba	6.83	3.16	2.84	83.53	3.65
Maritimes	5.10	6.21	0.89	57.57	30.24
Newfoundland	2.88	28.86	0.14	67.61	0.52
Nunavut	1.01	66.77	0.01	32.25	0.01
Canada	2.12	10.42	6.28	74.06	7.12

Table S3: Data corresponding to Fig 8c in main text. Air Quality (AQ) Alert Bulletins issued by MSC-ECCC, 2017-2023. Data representing the number of all AQ Alert bulletins issued throughout the period. Each AQ alert will have a minimum of 2 bulletins issued (issuance and termination), plus a number of continued bulletins based on the persistence of the event. Each AQ Alert's area of coverage varies in size based on the geographical extent of the AQ event.

	2017	2018	2019	2020	2021	2022	**2023**
Jan	122	97	53	32	45	79	**34**
Feb	77	61	67	87	52	43	**59**
Mar	50	129	366	49	102	90	**142**
Apr	20	66	68	0	20	10	**28**
May	31	53	108	33	104	19	**520**
Jun	9	31	185	48	160	64	**1412**
Jul	223	217	273	22	728	245	**932**
Aug	413	589	66	70	407	191	**979**
Sep	385	54	15	96	101	247	**762**
Oct	32	22	6	9	77	153	**72**
Nov	85	67	17	12	27	138	**55**
Dec	127	37	69	27	10	43	**21**
Total	1574	1423	1293	485	1833	1322	**5016**

Table S4. Ten largest wildfire evacuations on record, by number of evacuees[2]. Five of the ten largest evacuation events (by number of evacuees) since 1980 occurred in 2023.

Evacuation Date	Province/Territory	Location	Evacuees
2016-05-03	AB	Fort McMurray	88000
2003-08-18	BC	Kelowna	33050
2023-08-16	NT	Yellowknife	21720
2023-08-17	BC	West Kelowna	19809
2023-05-28	NS	Halifax	16400
2009-07-18	BC	West Kelowna	11000
2017-07-15	BC	Williams Lake	10753
2023-08-17	BC	Kelowna	9757
2023-05-05	AB	Edson	8414
1998-08-10	BC	Salmon Arm	8000

North American Drought Monitor

To evaluate general drought conditions in Canada, we used summary statistics from the North American Drought Monitor (NADM)[3]. The provided statistics consists of a monthly time series of drought classification for each province/territory. Classifications include D0 (Abnormally Dry), D1 (Moderate Drought), D2 (Severe Drought), D3 (Extreme Drought), D4 (Exceptional Drought). We retrieved data for the percentage area with conditions D0-D4, i.e., Abnormally dry or in drought.

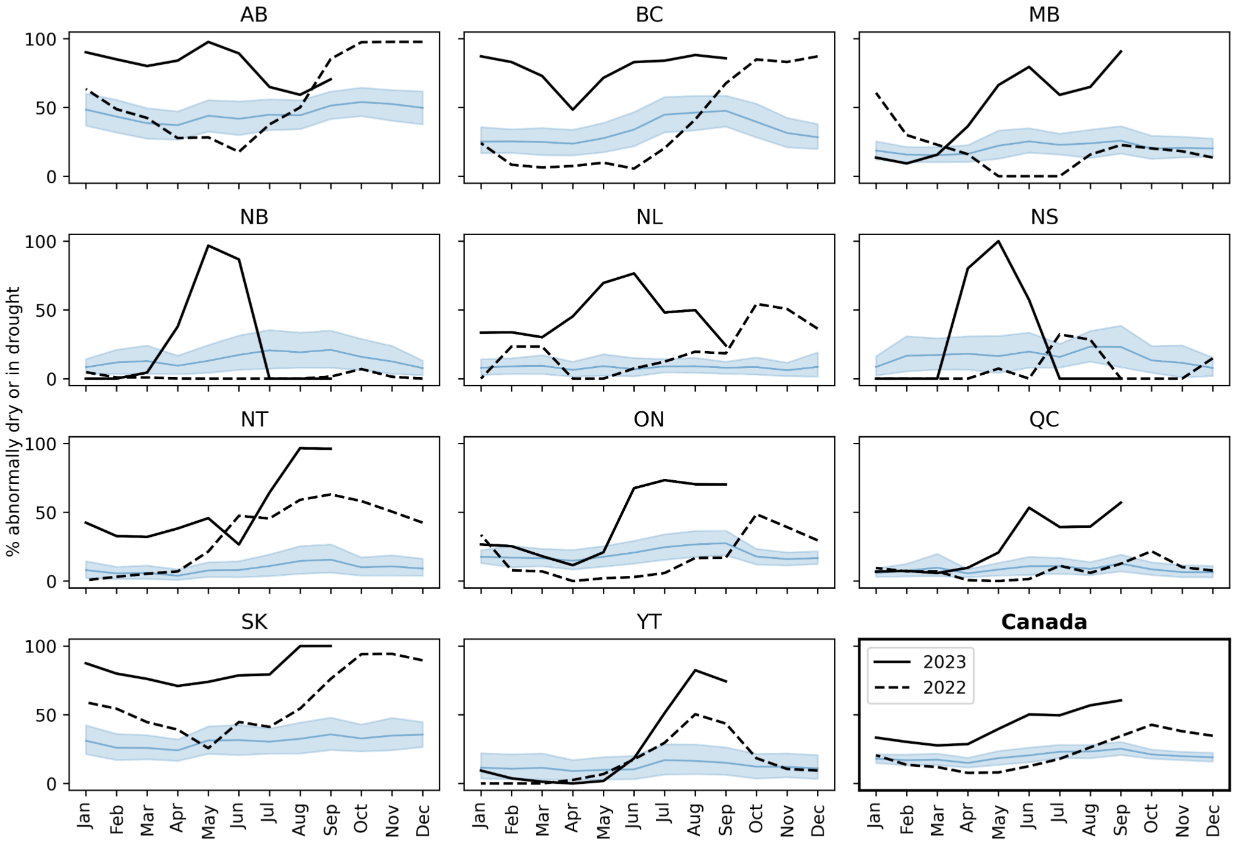

Fig S1: Monthly time series of percentage of each province or territory that is abnormally dry or in drought conditions for 2022 and 2023. The climatological mean (2002-2023) is shown by the blue line with 95% confidence intervals shaded. Data from the North American Drought Monitor (see methods). See Lawrimore et al 2002 for drought definitions[4].

Extreme Fire Weather

The 95th percentile of the Initial Spread Index (ISI) and Build-up Index (BUI), indices which are components (output indices) of the FWI System[5] showed similar regional and temporal trends to FWI_{95} in 2023 (Fig. 6 main text). Because of its dependence on wind speed, the ISI is related to wind-driven fire regimes, whereas the BUI - which depends on the Drought Code (DC) and Duff Moisture Code (DMC) - is related to drought-driven fire regimes.

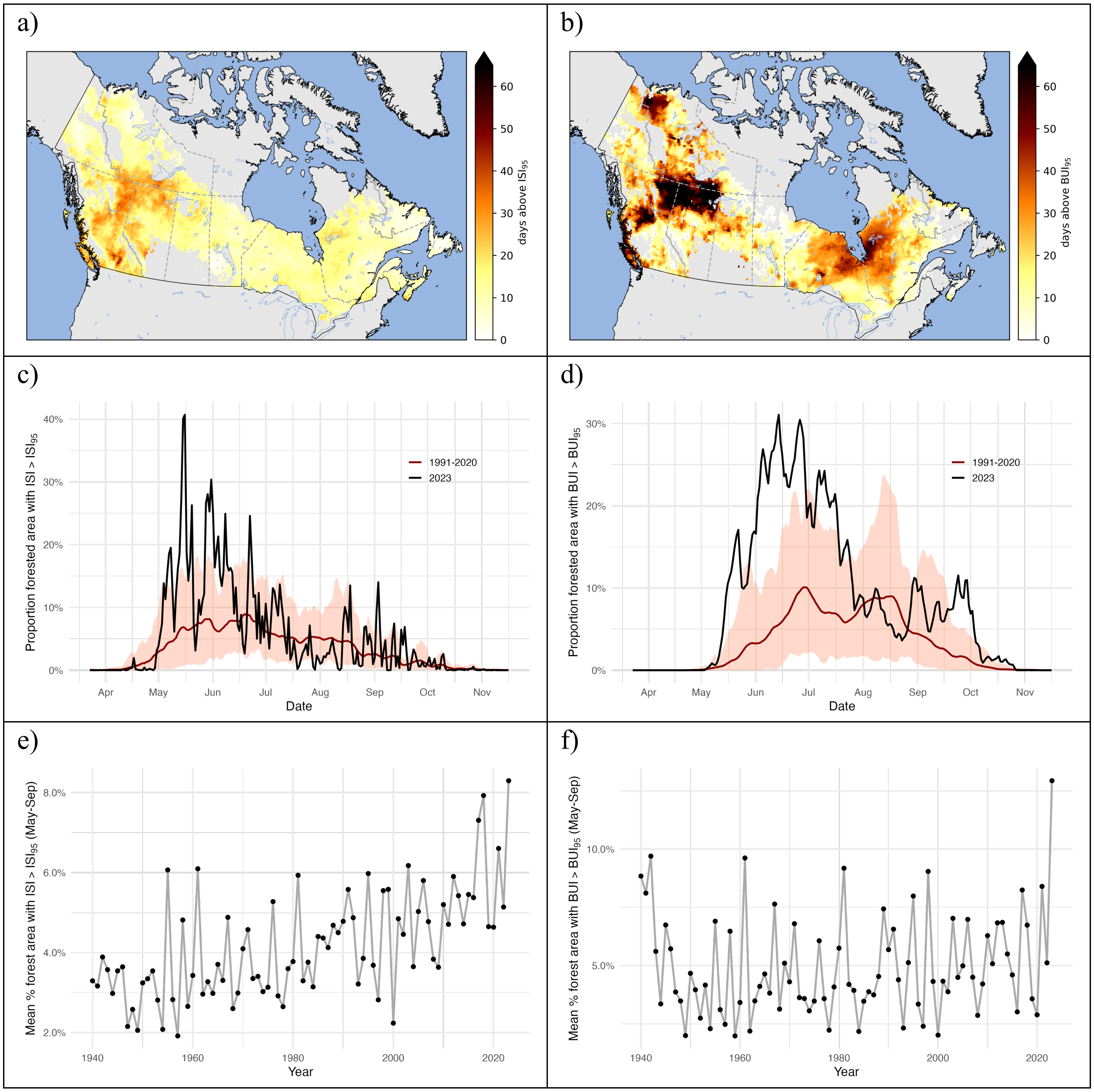

Fig. S2: a) Total number of days exceeding local 95th percentile of ISI for 2023; c) Total forested area exceeding local 95th percentile of ISI (black) compared with 5th and 95th percentiles (shaded) and mean (red) values for 1991-2020; e) Mean percent daily forested area exceeding local 95th percentile of ISI during fire season (May-Sept) for

each year (1940-2023); b) Total number of days exceeding local 95th percentile of BUI for 2023; d) Total forested area exceeding local 95th percentile of BUI (black) compared with 5th and 95th percentiles (shaded) and mean (red) values for 1991-2020; f) Mean percent daily forested area exceeding local 95th percentile of BUI during fire season (May-Sept) for each year (1940-2023).

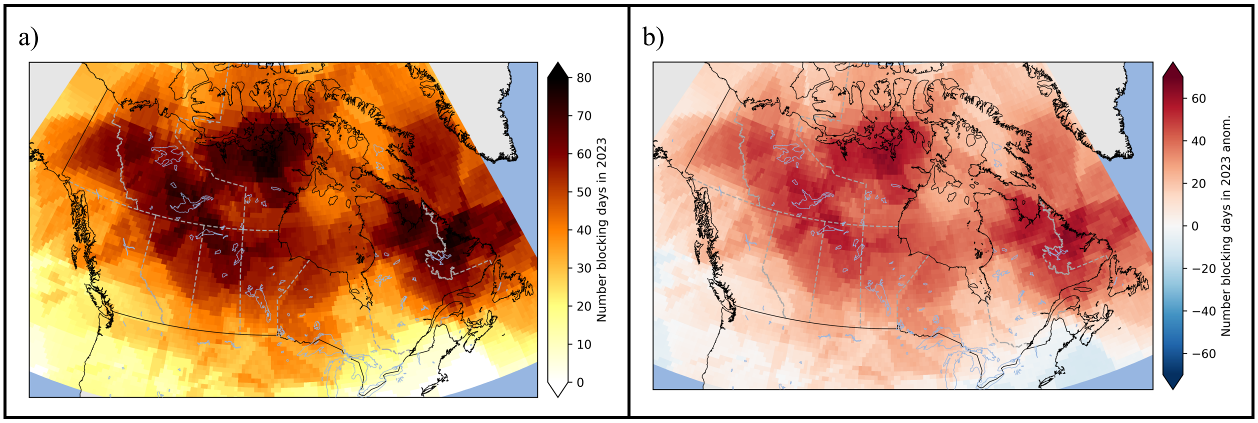

Fig. S3: a) The number of blocking days between April 1st and October 31st 2023; b) the anomaly in the number of blocking days between April 1st and October 31st 2023 relative to the baseline period (1991-2020). Here blocking days are defined as the number of days each year at each location with persistent positive anomalies in 500-hPa geopotential heights. See methods for details.

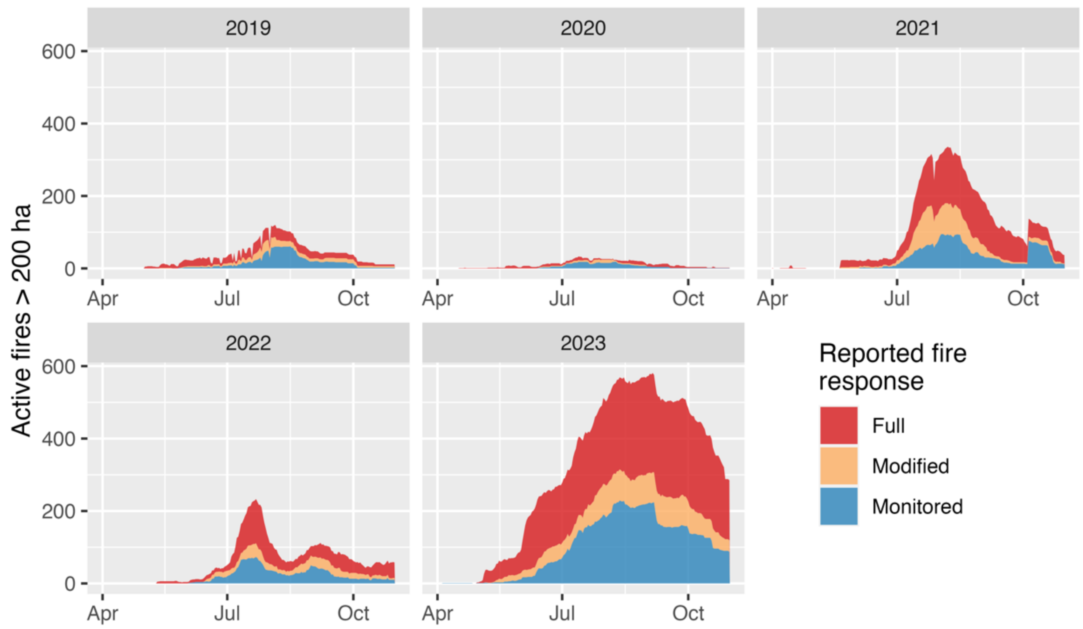

Fig. S4: Number of active fires > 200 ha for 2019–2023, by reported suppression action (fire response) using archived CIFFC fire agency data.

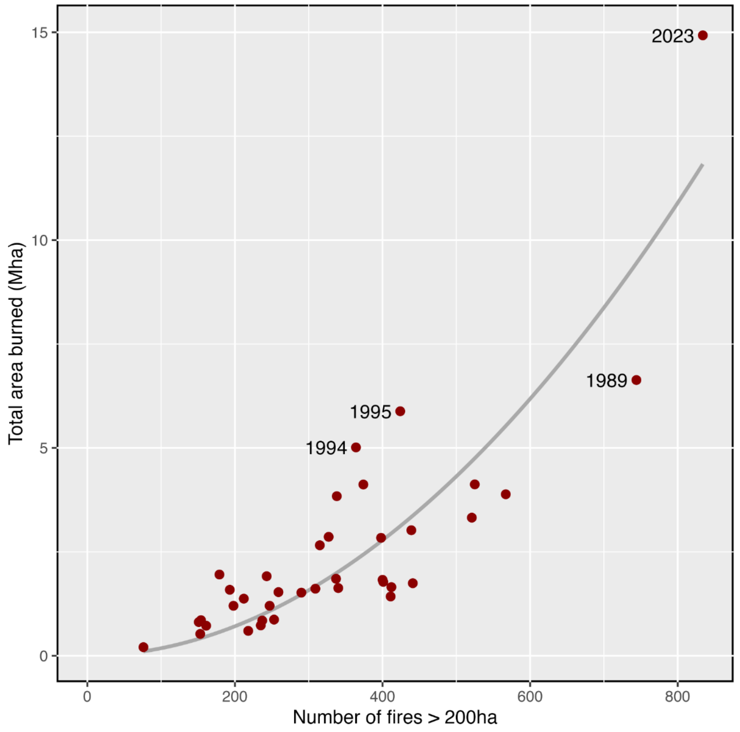

Fig. S5: Total annual area burned in Canada as a function of number of fires > 200ha using NBAC data (1986–2023). The four years with the greatest area burned are indicated. The relationship follows an approximate power law. Large fires > 200 ha account for 95% of area burned in Canada. In 2023, 2.6 times the average number of large fires burned about 7 times the average area.

Population exposure to wildfire smoke

We combined population data with the 2023 modeled surface $PM_{2.5}$ concentrations (see methods in main text) to determine population exposure to wildfire smoke during 2023. For regional exposure (province or territory) we used the gridded population downloaded from: https://sedac.ciesin.columbia.edu/data/collection/gpw-v4/sets/browse. For urban centers we used the Census Metropolitan areas from the Government of Canada open data portal: https://open.canada.ca/data/en/dataset/096b2a17-2755-40fe-b750-1c489c5e6b6a. For regional wildfire smoke exposure, we calculated a weighted mean of days of $PM_{2.5} > 27\mu gm^{-3}$ using the gridded population density as the weighting factor. For census metropolitan areas we extracted days of $PM_{2.5} > 27\mu gm^{-3}$ to each area. The resulting values represented the per capita number of poor air quality experienced in 2023 .

Table S5: Poor air quality days per capita for each province/territory of Canada, defined as the population weighted mean of days with $PM_{2.5}$ greater than $27\mu gm^{-3}$.

Region	Smoky days per capita
British Columbia	3.5
Alberta	17.1
Saskatchewan	10.6
Manitoba	7.9
Ontario	8.7
Quebec	6.6
New Brunswick	0.2
Yukon	1.7
Nunavut	0.4
Newfoundland and Labrador	0.7
Nova Scotia	0.1
Northwest Territories	43.6
Prince Edward Island	0
Canada	8

Table S6: Mean poor air quality days and maximum daily $PM_{2.5}$ for the 10 most populous census metropolitan areas.

Census metropolitan area	population	poor AQ days	max daily PM2.5
Toronto	6,202,225	14	129
Montreal	4,291,732	12	135
Vancouver	2,642,825	4	112
Ottawa - Gatineau	1,488,307	11	215
Calgary	1,481,806	18	103
Edmonton	1,418,118	21	219
Quebec	839,311	4	117

Winnipeg	834,678	10	70
Hamilton	785,184	11	127
Kitchener - Cambridge - Waterloo	575,847	7	97

Daily growth of ten largest wildfires in 2023.

We considered individual cases of the 10 largest mapped fires of 2023 by examining daily area burned, the corresponding mean daily FWI values and the daily precipitation at the location of the fire.

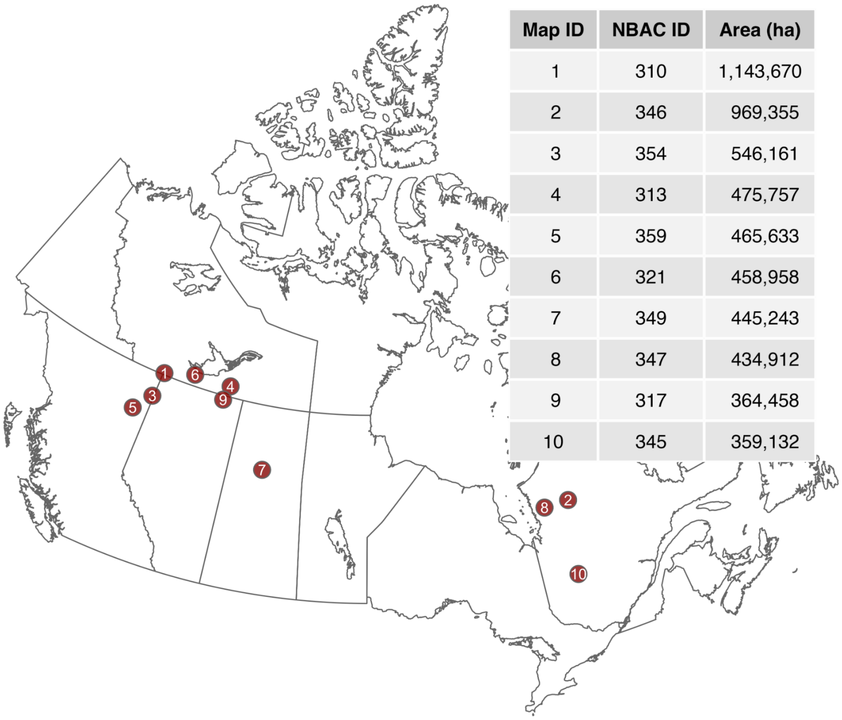

Map ID	NBAC ID	Area (ha)
1	310	1,143,670
2	346	969,355
3	354	546,161
4	313	475,757
5	359	465,633
6	321	458,958
7	349	445,243
8	347	434,912
9	317	364,458
10	345	359,132

Fig. S6: Location of the 10 largest wildfires that burned in Canada in 2023.

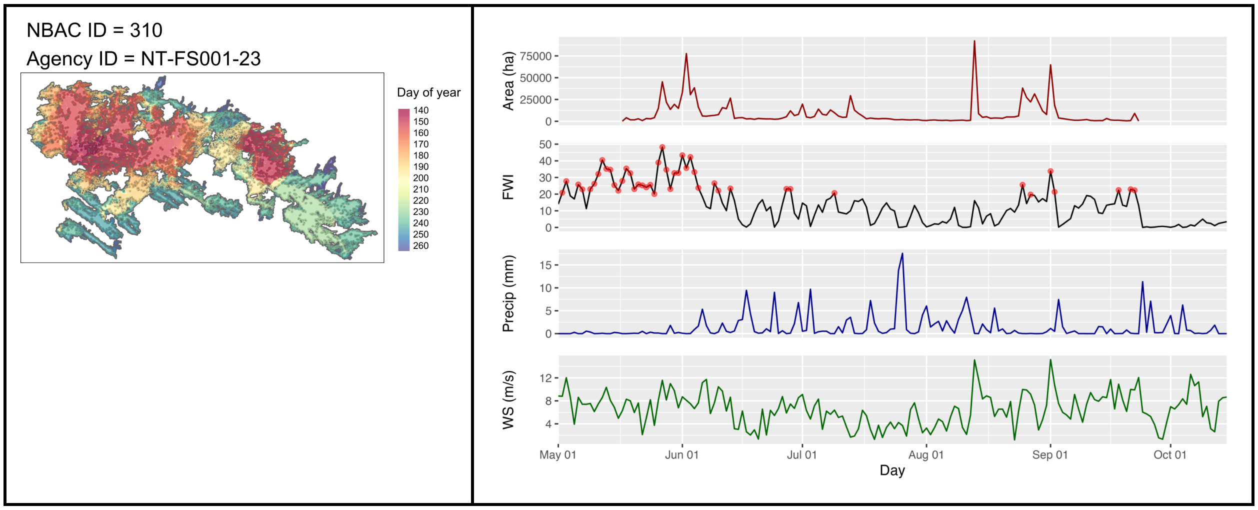

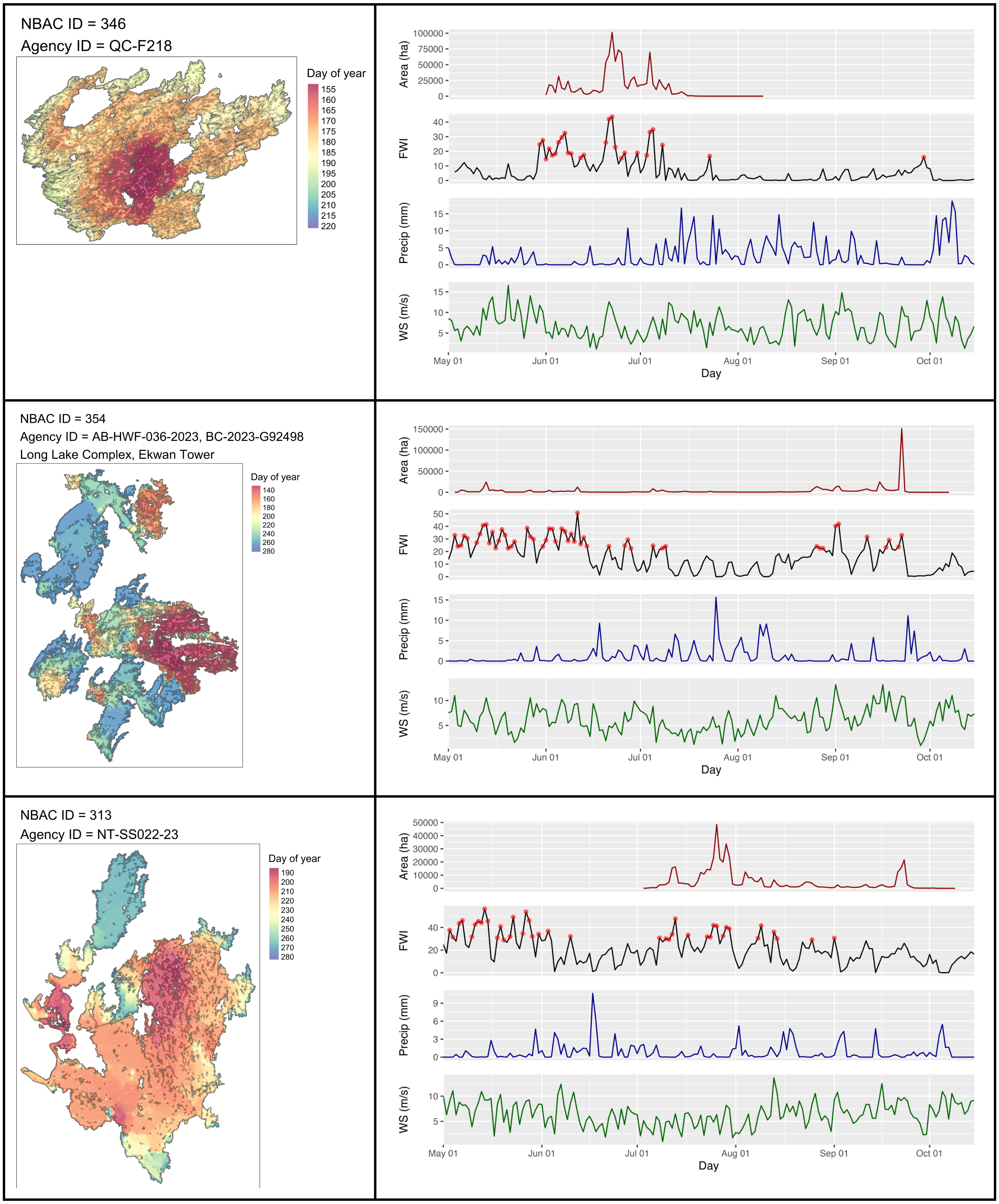
NBAC ID = 346
Agency ID = QC-F218
Day of year
155 160 165 170 175 180 185 190 195 200 205 210 215 220
Area (ha)
FWI
Precip (mm)
WS (m/s)
May 01 Jun 01 Jul 01 Aug 01 Sep 01 Oct 01
Day
NBAC ID = 354
Agency ID = AB-HWF-036-2023, BC-2023-G92498
Long Lake Complex, Ekwan Tower
Day of year
140 160 180 200 220 240 260 280
Area (ha)
FWI
Precip (mm)
WS (m/s)
May 01 Jun 01 Jul 01 Aug 01 Sep 01 Oct 01
Day
NBAC ID = 313
Agency ID = NT-SS022-23
Day of year
190 200 210 220 230 240 250 260 270 280
Area (ha)
FWI
Precip (mm)
WS (m/s)
May 01 Jun 01 Jul 01 Aug 01 Sep 01 Oct 01
Day

NBAC ID = 359
Agency ID = BC-G80280
Donnie Creek

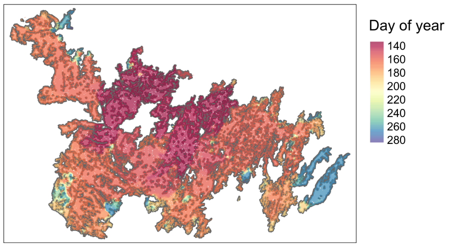

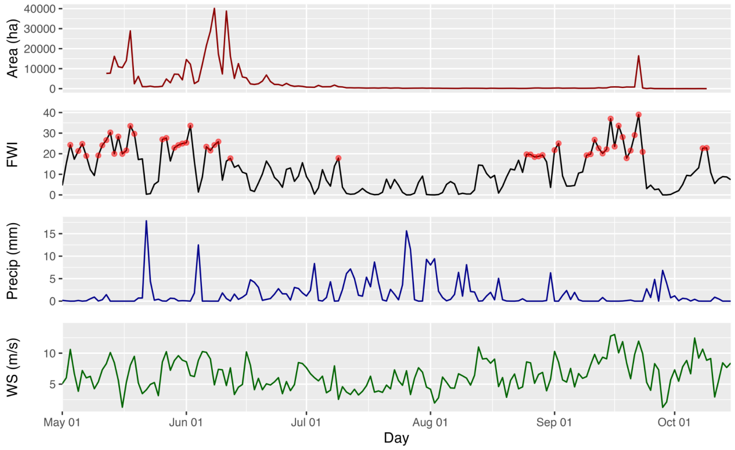

NBAC ID = 321
Agency ID = NT-SS052-23
Hay River

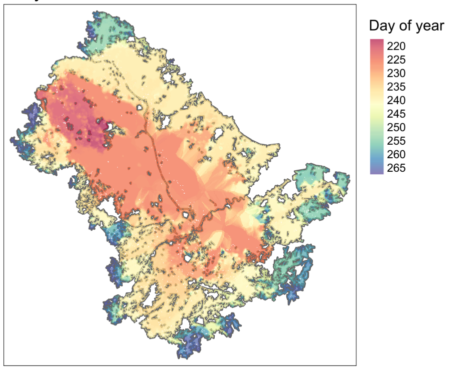

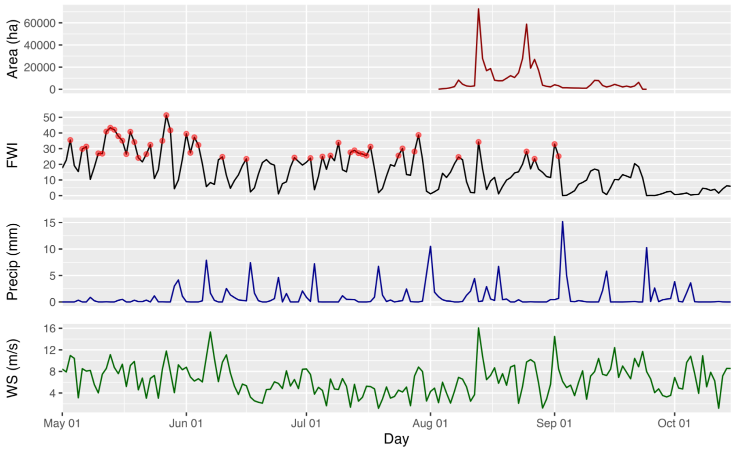

NBAC ID = 349
Agency ID = SK-23LX-SMITH

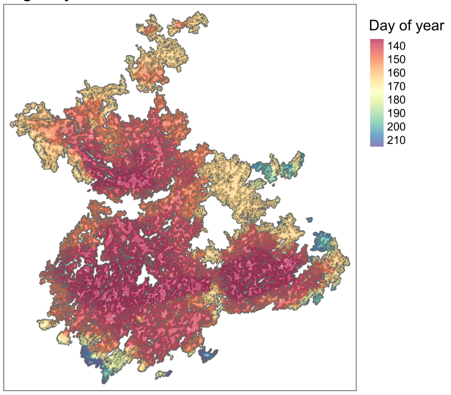

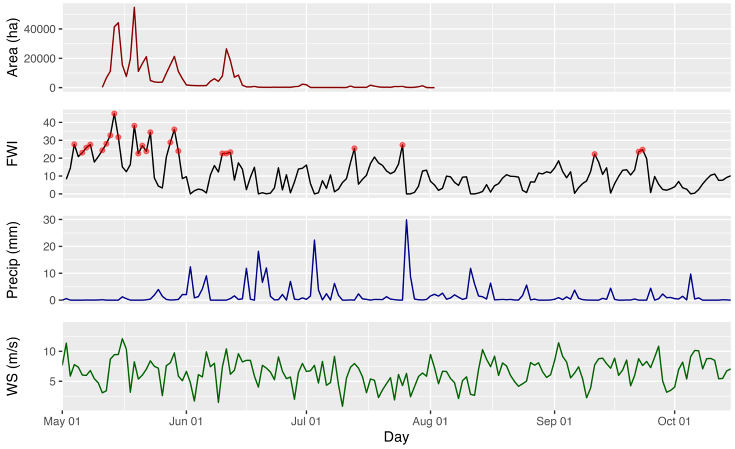

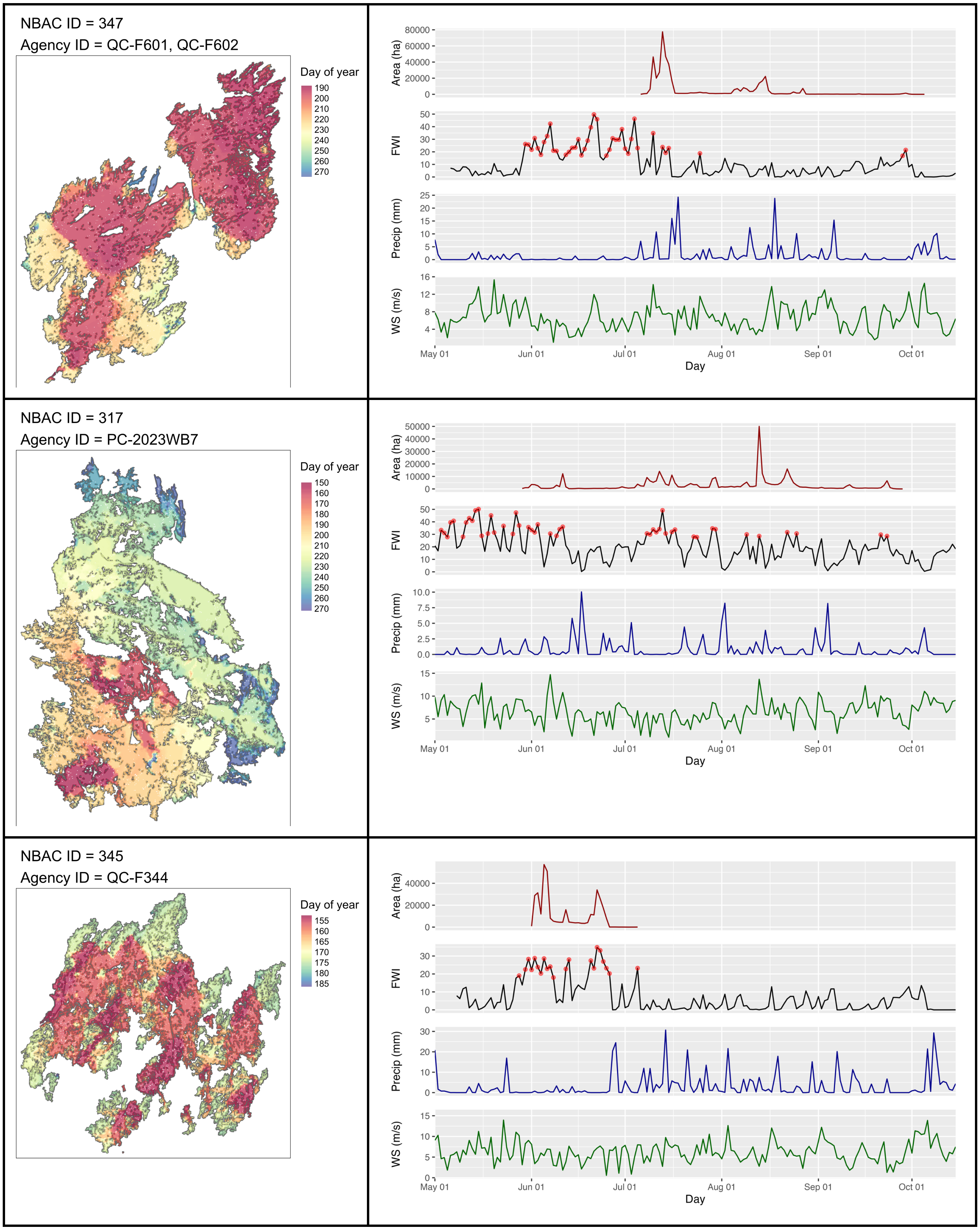

Fig. S7: Interpolated day of year of burning for the 10 largest wildfires that burned in Canada in 2023. Also shown for each fire is daily area burned, Fire Weather Index values at the fire location (with extreme fire weather days defined by days exceeding the 95th percentile of the local climatology fire season FWI, shown by red points), daily precipitation and 100m wind speed (mean values were taken over each fire perimeter).

Supplementary References

1. Guindon, L. et al., 2024. A new approach for Spatializing the CAnadian National Forest Inventory (SCANFI) using Landsat dense time series. Canadian Journal of Forest Research.

2. Christianson, A.C. et al. Wildland fire evacuations in Canada from 1980 to 2021. International Journal of Wildland Fire 33, WF23097 (2024)

3. National Drought Mitigation Center, University of Nebraska-Lincoln. "Statistics." North American Drought Monitor website. https://droughtmonitor.unl.edu/NADM/Statistics.aspx. Retrieved November 1st 2023.

4. Lawrimore, J., Heim, R.R., Svoboda, M., Swail, V. and Englehart, P.J. Beginning a new era of drought monitoring across North America. Bulletin of the American Meteorological Society, 83(8), pp.1191-1192 (2002).

5. Van Wagner, C. E. Development and Structure of the Canadian Forest Fire Weather Index System. Technical Report 35. Canadian Forest Service, Ottawa, Ontario, Canada (1987).
